# Small-sided games in volleyball: A systematic review of the state of the art

**DOI:** 10.5114/biolsport.2022.109960

**Published:** 2021-12-30

**Authors:** Henrique de Oliveira Castro, Lorenzo Laporta, Ricardo Franco Lima, Filipe Manuel Clemente, José Afonso, Samuel da Silva Aguiar, Alexandre Lima de Araújo Ribeiro, Gustavo De Conti Teixeira Costa

**Affiliations:** 1 Physical Education Department, Universidade Federal de Mato Grosso, Cuiabá, Brazil; 2 Universidade Regional Integrada do Alto Uruguai e das Missões, Santiago, Brazil; 3 Escola de Educação Física, Fisioterapia e Dança, Universidade Federal do Rio Grande do Sul, Porto Alegre, Brazil; 4 Escola Superior de Desporto e Lazer, Instituto Politécnico de Viana do Castelo, Viana do Castelo, Portugal; 5 Research Center in Sports Performance, Recreation, Innovation, and Technology, Portugal; 6 Instituto de Telecomunicações, Department of Covilhã, Covilhã, Portugal; 7 Centre for Research, Education, Innovation and Intervention in Sport, Faculty of Sport (CIFI2D), University of Porto, Porto, Portugal; 8 Centro Universitário do Distrito Federal, Brasília, Brazil; 9 Physical Education College, Universidade de Brasília, Brasília, Brazil; 10 Universidade Federal de Goiás, Goiânia, Brazil

**Keywords:** Team sports, Volleyball, Conditioned game, Drill-based games, Reduced game, Small-sided games

## Abstract

Studies on small-sided games (SSG) in team sports have increased in recent decades. However, the literature concerning this training strategy in volleyball is sparse. This study aims to summarize and analyse the scientific evidence on SSG in volleyball. For this purpose, electronic searches were conducted in August 2021 in PubMed, Scielo, ScienceDirect, Scopus, SPORTDiscus, and Web of Science databases. As result, a total of 22 studies (3 cross-sectional, 7 quasi-experimental, and 12 randomized controlled trial) that used SSG in volleyball were included in the qualitative synthesis after applying the eligibility criteria. Despite the few studies available for each outcome, our results suggest that the SSG can be used as a methodological resource for volleyball teaching and training of educational, recreational, and high-performance character. In conclusion, the use of SSG in volleyball is a pedagogical and training alternative with positive effects on populations with different levels of training (school and university students, recreational adult players, and athletes) considering instructional approaches, sport knowledge, participation in Physical Education classes, health markers, physical fitness, and physiological, psychological, and tactical-technical variables. However, more studies need to be carried out using SSG in volleyball in different contexts, with different manipulations and variables.

## INTRODUCTION

Small-sided games (SSG) are constrained game-based drills that change the structural dynamics of the formal match [1]. The design of these games is related to the management of task constraints [2], typically manipulating the number of players and their numerical relationships, area configuration, scoring method, permitted actions, strategic tactical actions, and/or training regimen [3–8]. Thus, SSG require players to adapt to novel game scenarios with different situational contexts [9, 10].

SSG are very popular in team sports, mainly because they increase the perception of the players for specific tactical/technical issues while some of these challenges promote variations in the physiological and physical stimuli [11], with possible consequences for biological adaptations in the medium to long term [12, 13] and the possibility may also help in improving specific technical skills and tactical behaviours [2, 3, 10]. Thus, SSG allow the identification of the skill level of the players, as well as proposing effectual interventions in the training process in a contextualized and situational way [14]. The utilization of SSG seems to be an effective tool, both in initiation [15] and in high performance [3]. However, due to the specificity of SSG, task constraints can act differently from sport to sport, particularly between invasion sports and net sports [16].

In this regard, volleyball is a team net ball sport with an intermittent type of effort and characterized by unpredictability [17]. Volleyball is a sport that requires different elements for its qualification, such as technique, tactics, and physical demands that favour aspects of the game [18–20], and involves a combination of repetitive jumps, multidirectional movements, and long, extended matches [21]. Because volleyball is a net sport, both teams play in separate courts, with the possession of the ball changing cyclically during the rally [22]. The performance of the game phases varies dynamically according to player characteristics (e.g., physical features, technical level, and maturity state), rule changes, environmental factors [23], and high-intensity court movement that occurs repeatedly during the training and matches [24]. Skill-based training is used to improve team sports performance [25], and volleyball players depend on well-developed physiological capacities and tactical and technical high-level skills [24].

Sarmento et al. [25] indicated in a review in soccer that SSG with lower player numbers lead to more technical actions performed per player, more consistent tactical behaviours, and increased physiological demands. SSG in volleyball are usually adopted to increase the number of contacts with the ball and facilitating and speeding up the adoption of specific techniques [8]. For example, in 3 vs. 3 SSG, the players have a higher average number of contacts with the ball per set than in 6 vs. 6 games (approximately 27.7 ball contacts more) [8]. Studies with young volleyball athletes [26–28] demonstrated improvements in technical and tactical demands.

In addition, the exercise intensity of SSG can be demonstrated through a player’s movement and/or physiological/perceptual responses. Many prescriptive variables can be controlled by the coach to influence the exercise intensity during SSG [29]. In soccer, the intensity of SSG is higher on larger pitches and with a smaller amount of players [30]. In this line, in physiological demands, Gabbet et al. [24] did not find a difference in junior athletes but, at the adult recreational level, Trajković et al. [31] found improvements in physiological markers using SSG in volleyball. However, the intensity of SSG can vary significantly, and the optimal design remains to be determined [32]. There is a lack of studies for a better discussion on the subject of intensity in SSG in volleyball.

One adaptation of the traditional sport of volleyball, used as SSG for school students, is mini-volleyball [33, 34]. Mini-volleyball is a modified game (the size of the court, the number of players, and net height are reduced) that increases the student’s participation, skill-level knowledge, decision-making, skill execution, and game performance [33]. Although mini-volleyball promotes more opportunities to participate and develop physical literacy in the participants [34], the tactical knowledge is not developed from this approach [33]. Studies involving mini-volleyball demonstrated an improvement in motivation, participation in Physical Education classes, socialization, and basics skills in children and adolescents [35, 36, 37].

From the point of view of the ecological approach [38–40, 41], players must train and adapt to the variability of actions in different contexts, in order to have effective evolutions when experiencing these training problems [42] and possibilities for action [38]. SSG in volleyball should be used to prepare the players for these considerable variability of actions that the context can provide, to involve them tactically, technically, physically, and psychologically according to the restrictions and environmental context [28, 31, 42]. Therefore, it is important that more studies are carried out with the evaluation of different parameters using SSG in volleyball.

Although volleyball represents one of the most popular ball sports in the world [43], and the studies on SSG in team sports have increased more in the last two decades [10], the literature involving this training structure in volleyball is sparse [10, 30, 44]. Recent systematic review studies involving SSG in team ball sports have been conducted in soccer [10, 25, 45, 46] and basketball [47], but to our knowledge no such synthesis has been done in volleyball.

Hence, the following questions are raised: What is the evidence concerning the utilization of SSG in volleyball? What are the research gaps that remain to be explored? A systematic review can provide a synthesis of the evidence and assess its quality, paving the way for future studies. Therefore, the present study aimed to summarize and analyse the state of the art of scientific evidence about small-sided games in volleyball.

## MATERIALS AND METHODS

### Preliminary settings

This study was reported according to the Preferred Reporting Items for Systematic Reviews and Meta-Analyses (PRISMA) [48].

The study design was done according to the PICOS strategy, in which the population (P) was defined as volleyball players, the necessary intervention (I) was small-sided games, comparison (C) was not required, the outcomes (O) of interest were those measured through small-sided games (e.g., physical tactical, among others), and like the comparison, there were no limitations on study design (S).

### Search methods for identification of studies

For searches in databases, the following combination was used with the Boolean operators (and, or) in all fields, using Medical Subject Headings (MeSH terms) and text words (free descriptors) of interest in each database: (volleyball OR volleyballs) AND (“conditioned game” OR “conditioned games” OR “drill-based game” OR “drill-based games” OR “play format” OR “play formats” OR “reduced game” OR “reduced games” OR “sided-game” OR “sided-games” OR “small-sided and conditioned game” OR “small-sided and conditioned games” OR “small-sided game” OR “small-sided games” OR ssg OR ssgs OR “mini-volleyball” OR “mini-volleyballs”). This search was conducted on August 19, 2021, in the following databases: PubMed, Scielo, ScienceDirect, Scopus, SPORTDiscus, and Web of Science. There were no restrictions regarding the year of publication or study type, and no search filter was used. Also, manual research was conducted on the bibliographies of all included studies in full-text screening.

### Eligibility criteria

The eligibility criteria (inclusion and exclusion) can be observed in Table 1.

**TABLE 1:** Eligibility criteria.

Criteria	Inclusion criteria	Exclusion criteria
Population	Indoor volleyball players from any sex, age-group (children/adolescents, adults, and the older adults), skill, or competitive level (educational, recreational, and high performance/athlete).	Non volleyball players (e.g., beach volleyball; soccer; basketball; rugby), or clinical populations
Intervention	Players exposed to small-sided games (e.g., any format of play within 1 vs. 1 and 6 vs. 6). Including mini-volleyball. For the present study, small-sided games were defined as widely used drill-based exercises that simplifies the dynamic of real team sport game while keeping the main properties of the match [1].	Any drill that does not present a dynamic of the game proper of volleyball and does not characterize a format of play (e.g., analytical drills, positional drills).
Comparator	No comparisons required.	Not applicable.
Outcome	(i) Tactical (ii) Technical (iii) Physical (iv) Physiological (v), Health markers (vi) Psychological, and (viii) Instructional approaches	Not applicable.
Study design	No restrictions with regard to study design. In case of more than one time-point, both (pre and post) will be considered.	Letters to editors, trial registrations, proposals for protocols, editorials, book chapters, reviews, and conference abstracts.
Language	Only original and full-text studies written in English, Portuguese, Spanish, Italian, and French.	Not applicable.

### Study selection

Two independent authors performed the removal of duplicates and initial screening (titles and abstracts) of the results identified by the search strategy. This process was carried out on the platform Rayyan (http://rayyan.qcri.org). Divergence in study selection was resolved by consensus between the initial reviewers, with no need for a third author. After initial screening, a full-text reading of the potentially eligible studies was done by the same authors, also independently, and without the need for a third author.

### Data extraction

Two review authors independently extracted the primary studies’ data using standard data extraction in Microsoft Excel 2016 software (version 16.0.13901.20148 for Windows) to collect the following details: study (last name of first author and year of publication), objective, sample, level of training, game design manipulating, variables and findings. This extraction was checked by a third author.

### Methodological quality

Two independent authors assessed the methodological quality of the included studies using the Appraisal tool for Cross-Sectional Studies – AXIS [49] for cross-sectional studies, the PEDro scale for randomized trials, and the Joanna Briggs Institute (JBI) critical appraisal checklist for quasi-experimental studies. These evaluations were checked by a third author.

AXIS includes 20 items that must be answered with “yes”, “no” or “I don’t know”. These items report the methodological quality and the risk of bias in cross-sectional studies. In detail, items 1 and 2 assess suitability for the study design, items 3 to 7 assess the selection bias, items 8 and 9 assess the measurement bias, items 10 to 16 assess the reporting bias, and items 17 to 20 assess the presence of possible confounding factors that can hinder the interpretation of the study results [49].

The PEDro scale consists of 11 criteria (random allocation; concealed allocation; baseline comparability; blind subjects; blind therapists; blind assessor; adequate follow-up; intention-to-treat-analysis; between groups comparisons; point estimates and variability), which receives either a “yes”, or “no” rating. Although the PEDro scale has 11 criteria, the first criteria are not used in the calculation, so the maximum PEDro score is 10 points. Trials with a PEDro score ≥ 6 points were classified as high-quality, while trials with a PEDro score < 6 points were classified as low quality. The studies were assessed with the Brazilian-Portuguese version of the PEDro scale.

The JBI critical appraisal checklist for quasi-experimental studies includes 9 items that must be answered with “yes”, “no”, “unclear” or “not applicable”. These items (Is it clear in the study what is the ‘cause’ and what is the ‘effect’?; Were the participants included in any similar comparisons?; Were the participants included in any comparisons receiving similar treatment/care, other than the exposure or intervention of interest?; Was there a control group?; Were there multiple measurements of the outcome both before and after the intervention/exposure?; Was follow-up complete and if not, were differences between groups in terms of their follow-up adequately described and analysed?; Were the outcomes of participants included in any comparisons measured in the same way?; Were outcomes measured in a reliable way?; Was appropriate statistical analysis used?) report the methodological quality in non-randomized studies.

## RESULTS

**FIG. 1:**
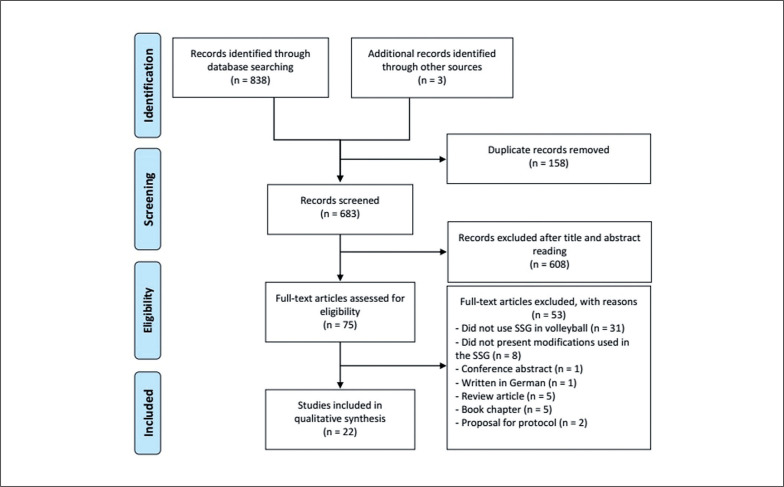
PRISMA Flowchart.

The electronic search retrieved 838 documents, of which 158 were excluded as duplicates. Also, before screening by titles and abstracts, another 3 documents not identified in the electronic searches were indicated by a specialist and added to the references for screening, totalling 683 documents. Of these, 608 were excluded through screening of the title and abstract, and 53 were excluded by full-text reading (reasons for exclusion are described in the flowchart). Thus, 22 studies [8, 24, 27, 28, 31, 35–37, 43, 50–62] were included in the qualitative synthesis (systematic review) after applying the eligibility criteria. Figure 1 shows the flowchart and screening of the studies included in the qualitative synthesis.

### Characteristics of the included studies

The studies had a total of 1309 participants of both genders (412 female, 428 male, 278 not specified in the study, and 191 uncertain). It is important to highlight that the 191 participants said to be uncertain is due to a possible typewriting error in the studies in which they take part [37, 43]. Thus, instead of putting the numbers of female and male participants, we decided to report only the main number of participants mentioned in these studies. The ages of participants ranged between 9 [35] and 44 [31] years. The sample sizes ranged between 12 [27] and 252 [62] participants. The game design manipulation ranged from the 1 vs. 1 [35, 36, 57, 60] to 5 vs. 5 [24, 36, 37, 52] players. In some cases, variations of the 9 m² to 128 m² size of the court were added [27, 28, 31, 37, 43, 50, 53, 55, 58, 59, 61]. The training level included recreational participants [31], school students [8, 35, 36, 37, 43, 53, 54, 56, 57, 60, 61, 62], university students [51], and elite athletes [24, 27, 28, 50, 52, 55, 58, 59]. Additional characteristics of the included studies (study design, objective, variables, and findings) can be consulted in Table 2.

**TABLE 2:** Characteristics of the included studies (n = 22).

Study	Study design	Objective	Participants	Level of training	Game design manipulation	Variables	Findings
Stojanović et al. [8]	Randomized controlled trial	This study aims to determine the effects of a 16-week program of skill-based exercises and SSG related to volleyball and regular PE curriculum on changes in body composition parameters.	90 school students (47 boys and 43 girls; age 13 ± 6 years).	School students	Players: - 2 vs. 2 - 3 vs. 3 - 4 vs. 4	Anthropometric characteristics and body composition (body height, body mass, skinfold)	Skill-based exercises and SSG program: decrease of skinfold thickness (both genders) and body fat tissue (boys), and an increase of muscle tissue (both genders). Regular PE curriculum: increased body fat tissue (both genders) and muscle tissue (girls), while a decrease in muscle tissue was recorded among the boys.
Gabbett et al. [24]	Quasi-experimental	Effect of a skill-based training program on measurements of skill and physical fitness in talent-identified volleyball players.	26 junior volleyball players (gender not informed; age 15.5 ± 0.2 years).	Elite players (5 ± 1 year of experience)	Players: - 3 vs. 3 - 5 vs. 5	Anthropometric characteristics (height, standing-reach height, body mass, and sum of 7 skinfolds), physical fitness (lower and upper-body muscular power, speed, agility and maximal aerobic power), and skill (passing, setting, serving, and spiking technique and accuracy)	Skill-based conditioning improves technical skill (spiking and passing), accuracy (spiking, setting, and passing), and physical fitness (speed and agility).
Rocha et al. [27]	Cross-sectional	Compare the tactical-technical behavior between two distinct situations of reduced volleyball games.	12 youth volleyball players (male; age 16.7 ± 1.5 years)	Elite players (3.2 ± 1.2 years of experience)	Players: - 2 vs. 2 Court size: - 3 × 3 m - 2 × 4.5 m.	Tactical-Technical behaviour	2 vs. 2 in 2 × 4.5 m: best adjustment and DM index in reception and attack; 2 vs. 2 in 3 × 3 m: best efficacy index in reception.
Rocha et al. [28]	Cross-sectional	Compare the tactical and technical behavior of beginner players in volleyball side-out between four distinct situations of reduced volleyball games with different area/players ratio.	16 youth volleyball players (male; age 12.2 ± 0.5 years).	Novices (1.2 ± 0.8 years of experience)	Players: - 2 vs. 2 Court size: - 3 × 3 m - 4 × 4 m - 4.6 × 4.6 m - 5.2 × 5.2 m	Tactical-Technical behaviour	2 vs. 2 in 3 × 3 m: best adjustment index; 2 vs. 2 in others: best efficiency index; 2 vs. 2 in 4.6 × 4.6 m and 5.2 × 5.2 m: high DM scores.
Trajković et al. [31]	Randomized controlled trial	Determine the effects of recreational SSG volleyball on health markers and physical fitness in the middle-age population.	26 adults middle-age (male; age 44.7 ± 6.3 years).	Recreational	Players: - 3 vs. 3 - 4 vs. 4 Court size: - 16 × 8 m	Health markers (blood collection) and physical fitness (heart rate, rate perceived exertion, and physical activity enjoyment scale)	SSG recreational volleyball: decrease in some risks factors (LDL, resting HR, and systolic BP) and better results in cardiovascular fitness
Millán and Borda [35]	Quasi-experimental	Develop and implement a teaching program of mini-volleyball based on ludic and adapted to school.	48 school students (24 boys and 24 girls; between 9–11 years old).	School students	Players: - 1 vs. 1 - 2 vs. 2 - 3 vs. 3 - 4 vs. 4	Knowledge and learning of mini-volleyball	The mini-volleyball program had significant impact on the knowledge and learning game situations, the motivation, and basic technical skills.
Batez et al. [36]	Randomized controlled trial	Investigate the effects of the TGfU Mini-volleyball Unit program implemented in PE classes on volleyball skills and enjoyment in secondary school students	54 adolescent students (18 girls and 36 boys) divided in experimental group (n = 28; 15.5 ± 0.7 years old) and control group (n = 26; 15.7 ± 0.6 years old).	Adolescent school students	Players: - 1 vs. 1 - 2 vs. 2 - 3 vs. 3 - 4 vs. 4 - 5 vs. 5	Volleyball skills (service, overhead and forearm passing, and setting) and the sport enjoyment.	TGfU Mini-volleyball Unit program improved overhead and forearm passing skills and better enjoyment in experimental group compared to control group.
Ningrum et al. [37]	Randomized controlled trial	Verify the effectiveness of SSG learning model on the mobile phone application to increase student participation in PE	84 high school students (Experimental group: n = 42; Control group: n = 42).	High school students	Players: - 3 vs. 3 - 4 vs. 4 - 5 vs. 5 Court size: - 5 × 10 m	Forearm skills and student participation in PE	Students who experienced the SSG had significantly higher and moderate PE activity levels, friendship-approach and friendship-avoidance goals; Improvement volleyball forearm pass skill.
Trajković et al. [43]	Randomized controlled trial	Determine the effect of after-school small-sided volleyball on aggression and physical fitness in students.	107 school students between 14–16 years old.	School students	Players: - 3 vs. 3 - 4 vs. 4 Court size: - 9 × 4.5 m - 12 × 6 m	Physical fitness (medicine ball, vertical jump and yo-yo intermittent recovery level 1 test) and psychological (aggression)	After-school SSG volleyball: decrease in aggression and better results in physical fitness; girls showed larger reductions in aggression compared to boys with similar improvements in physical fitness.
Trajković et al. [50]	Randomized controlled trial	Determine the effects of SSG training on precision in young female volleyball players.	42 youth volleyball players (female; age 11.2 ± 1.1 years).	Elite players (2.2 ± 1.1 years of experience)	Players: - 2 vs. 2 - 3 vs. 3 Court size: - 7 × 3 m - 12 × 6 m	Skill accuracy (passing, setting, serving)	Small-sided games training was better in all parameters of accuracy (overhead pass, forearm pass, setting, serving, and serving under fatigue) compared to instructional training.
Broek et al. [51]	Quasi-experimental	Investigated the DM process of three instructional groups in practical courses in volleyball among university students.	122 university students (69 male; mean age 19.71, and 53 females; mean age 19.55).	University players	Players: - 4 vs. 4	DM and Instructional approaches	Tactical awareness: all students ameliorated after five lessons (posttest) and this effect persisted overtime after six weeks (retention test). Tactical awareness: male higher than female students. Student-centered instruction group with tactical questioning: improved volleyball specific tactical knowledge
Gabbett [52]	Randomized controlled trial	Investigate the specificity of skill-based conditioning games and compare the effectiveness of skill-based conditioning games and instructional training for improving physical fitness and skill in volleyball players.	25 junior volleyball players (12 boys and 13 girls; age 15.6 ± 0.1 years)	Elite players (experience not informed)	Players: - 5 vs. 5 - 5 vs. 4 - 5 vs. 3	Anthropometric characteristics (height, standing reach height, and body mass), physical fitness (speed, lower-body muscular power, agility and maximal aerobic power), and skill (passing, setting, spiking and serving)	Skill-based conditioning: stimulus the physiological demands of competition and improves the physical fitness (speed, vertical jump, spike jump, agility, upper-body muscular power, and maximal aerobic power); Instructional training: improves the technical skill (spiking and passing) and accuracy in all skill tasks (spiking, serving, setting, and passing).
Gil Arias et al. [53]	Quasi-experimental	Analyze the effect of manipulating different task constraints, such as play space, net height, and the number of participants on decision-making and efficacy in the attack action in volleyball.	1 teacher (male) and 22 school students (12 boys and 10 girls; age 12.5 ± 0.9 years).	School students	Players: - 3 vs. 3 - 4 vs. 4 Court size: - 6 × 6 m - 4.5 × 4.5 m Net height: - 2.10 m - 2.18 m	DM and skill demands (Attack Efficacy)	DM and attack efficacy: all participants improved between the pre-test and post-test measures.
Kim [54]	Quasi-experimental	Describe how improving a teacher’s content knowledge changes his teaching practices and its subsequent effects on student learning during a middle school volleyball instructional unit.	1 teacher (male) and 24 school students (12 boys and 12 girls)	School students	Players: - 3 vs. 3 - 4 vs. 4	SSG were used to enhance the cognitive understanding of content	Use of task progressions, integrated skill practices, small games, and adaptations of content, and diverse repertoires of verbal instruction after developing knowledge of the content: impacted the students’ game performance and involvement as well as a cognitive understanding of content.
Krističević et al. [55]	Randomized controlled trial	Determine the effects of game-based training on accuracy in adolescent volleyball players.	42 adolescent volleyball players (male; age 16.2 ± 1 years).	Elite players (5 ± 1 year of experience)	Players: - 3 vs. 3 - 4 vs. 4 Court size: 9 × 4.5 m	Skill accuracy (pass, setting, and serving)	No significant interaction between game-based training and instructional training; Game-based training (pre vs. post): improves serving and overhead pass; Instructional training (pre × post): improves serving, forearm pass, and setting.
Verscheure & Amade-Escot [56]	Cross-sectional	The study investigates how the attack is learned in volleyball according to gender through a design experiment within a didactic research methodology.	2 teachers (male) and 16 school students (10 boys and 6 girls; mean age not informed)	School students	Players: - 2 vs. 2	Skill (attack) in relation to the gender	Students do not develop the same nature and degree of understanding and performing the attack in volleyball due to the differentiated ways in which the didactical interactions evolve during formal lessons in PE.
Mahedero et al. [57]	Quasi-experimental	Examine the effects of student skill level on knowledge, DM, skill-execution and game performance in a mini-volleyball Sport Education season.	48 eight-grade students (23 boys and 25 girls), divided in higher or lower skilled group.	School students	Players: - 1 vs. 1 - 2 vs. 2 - 3 vs. 3 - 4 vs. 4	Student skill level on Knowledge (written test), DM (Individual interviews), skill execution and game performance (GPAI).	Improvements of the highest and lowest skilled students were less significant than those of more moderate levels. This outcome, accompanied by a lack of general improvement in skill execution.
Gjinovci et al. [58]	Randomized controlled trial	Compare effects of 12-week plyometric and volleyball-skill-based training on specific conditioning abilities in female volleyball players.	41 female adult volleyball players (age 21.8 ± 2.1 years; 1.76 ± 0.06 cm; 60.8 ± 7.0 kg)	Elite players	Players: - 3 vs. 3 - 4 vs. 4 Court Size: - 9 × 4.5 m	Anthropometrics, power and speed of lower and upper limbs.	Plyometric training: decrease in body mass (0.3% changes between pre- and post-measurement), and improvement in sprinting capacity (8% changes). Plyometric and skill-based training: improvement in jump and throwing capacities (plyometric training greater than skill-based training).
Idrizovic et al. [59]	Randomized controlled trial	Compare the effects of skill-based- and plyometric-conditioning on fitness parameters in female junior volleyball players	47 female junior players (age 16.6 ± 0.6 years).	Elite players	Players: - 3 vs. 3 - 4 vs. 4 Court Size: - 9 × 4.5 m	Anthropometrics, power and speed of lower and upper limbs, and flexibility of the hamstrings and lower back.	Plyometric training: improvement jump and throwing capacities, anthropometric characteristics, flexibility, and sprinting abilities. Skill-based training: improvement jump and throwing capacities, and flexibility.
Mahedero et al. [60]	Quasi-experimental	Explore any differences in game performance variables and knowledge among a cohort of high school students across a 12-lesson mini-volleyball Sport Education unit of study.	126 high school students (66 boys and 60 girls; average age = 16.6 years), divided in higher or lower skilled group.	High school students	Players: - 1 vs. 1 - 2 vs. 2 - 3 vs. 3 - 4 vs. 4	Quantitative: DM, skill execution, game performance, game involvement, and game knowledge. Qualitatively: (a) experts’ analysis of students’ game performance, and (b) students’ and teachers’ perceptions of students’ performance.	More competent in: Game play, more knowledge technique, sport’s rules, tactical awareness, and general game knowledge; By skill level had no impact on gains in game performance variables and knowledge.
Sgrò et al. [61]	Randomized controlled trial	Verify the effects of a tactical games model instructional plan on game-play volleyball performances of elementary school students, taking into account their skill level.	39 School students (18 girls and 21 boys; Average age = 8.9 years).	School students	Players: - 4 vs. 4 Court Size: - 6 m × 12 m Net height - 1.60 m	Volume of play index, efficiency index, and performance score.	Lower-skill-level students: reached or exceeded the scores of high-skill-level students at the pre-test, and large grater improvements in the pre vs. pos-test compared to high-skill-level students; Large improvement in the overall scores for each index in the pre vs. post-test.
Sujarwo et al. [62]	Randomized controlled trial	Develop mini-volleyball learning models to habituate character values (specifically discipline, cooperation, and hard work) for elementary school students.	30 PE teachers (32 ± 3.1 years old) and 252 students (11 ± 0.8 years old)	School students	Players: - 4 vs. 4	Habituate student characters (discipline, cooperation, and hard work)	The mini-volleyball learning model allowed the students have characteristics of discipline, cooperation, and hard work

SSG: Small-Sided Games; DM: Decision-Making; TGfU: Teaching Game for Understanding; PE: Physical Education.

### Methodological quality

Table 3 shows the assessments of methodological quality of the cross-sectional studies. Two studies were deemed to have a high methodological quality [27, 28] and only one study [56] had a low methodological quality.

**TABLE 3:** Methodological quality of the included cross-sectional studies, measured by the AXIS tool.

Study	Introduction	Methods										Results					Discussion		Other	
	1	2	3	4	5	6	7	8	9	10	11	12	13	14	15	16	17	18	19	20
Rocha et al. [27]	Y	Y	N	Y	Y	Y	N	Y	Y	Y	Y	Y	N	N	Y	Y	Y	N	N	Y
Rocha et al. [28]	Y	Y	N	Y	Y	Y	N	Y	Y	Y	Y	Y	N	N	Y	Y	Y	N	N	Y
Verscheure & Amade-Escot [56]	N	Y	N	Y	DK	DK	N	Y	Y	N	Y	Y	N	N	Y	Y	Y	Y	N	N

Y: Yes; N: No; DK: Don’t know.

Table 4 shows that two-thirds (eight studies) of randomized controlled trials had high methodological quality [8, 31, 36, 43, 50, 52, 55, 59], while only four had low methodological quality [37, 58, 61, 62].

**TABLE 4:** Methodological quality of the included randomized trials, measured by the PEDro scale.

Study	1	2	3	4	5	6	7	8	9	10	11	Total
Stojanović et al. [8]	1	1	0	1	0	0	0	1	1	1	1	6
Trajković et al. [31]	1	1	0	1	0	0	0	1	1	1	1	6
Batez et al. [36]	1	1	0	1	0	0	0	1	1	1	1	6
Ningrum et al. [37]	0	1	0	0	0	0	0	1	1	1	1	5
Trajković et al. [43]	1	1	0	1	0	0	0	1	1	1	1	6
Trajković et al. [50]	1	1	0	1	0	0	0	1	1	1	1	6
Gabbett et al. [52]	1	1	0	1	0	0	0	1	1	1	1	6
Krističević et al. [55]	1	1	0	1	0	0	0	1	1	1	1	6
Gjinovci et al. [58]	1	1	0	0	0	0	0	1	1	1	1	5
Idrizovic et al. [59]	1	1	0	1	0	0	0	1	1	1	1	6
Sgrò et al. [61]	1	1	0	0	0	0	0	1	1	1	1	5
Sujarwo et al. [62]	0	1	0	0	0	0	0	1	0	1	1	4

The quasi-experimental studies mostly (six studies) had high methodological quality [24, 51, 53, 54, 57, 60], as shown in Table 5.

**TABLE 5:** Methodological quality of the included non-randomized trials, measured by the JBI critical appraisal checklist for quasi-experimental studies.

Study	1	2	3	4	5	6	7	8	9
Gabbett [24]	Y	Y	N	N	Y	Y	NA	Y	Y
Millán and Borda [35]	N	N	N	N	Y	Y	NA	Y	N
Broek et al. [51]	Y	Y	N	N	Y	Y	NA	Y	Y
Gil Arias et al. [53]	Y	Y	N	N	Y	Y	NA	Y	Y
Kim [54]	Y	Y	N	Y	Y	Y	Y	Y	Y
Mahedero et al. [57]	Y	Y	N	N	Y	Y	NA	Y	Y
Mahedero et al. [60]	Y	Y	N	N	Y	Y	NA	Y	Y

Y: Yes; N: No; NA: Not Applicable

### SSG in volleyball: The state of the art Cross-sectional studies

One cross-sectional study analysed skill in school students [56]. This same study analysed the difference between gender [56] in school students. Another two studies analysed tactical-technical behaviour [27, 28] in elite players [27] and novices [28]. Therefore, although we found three studies, when analysed for a specific population or outcome (e.g., school or university students, or elite players), we did not find even two studies to compare them.

### Randomized controlled trials

Four studies analysed anthropometric characteristics [8, 52, 58, 59] in elite players [52, 58, 59] and school students [8], three studies analysed physical fitness [31, 43, 52] in elite players [52], school students [43] and recreational athletes [31], three studies analysed skill [50, 52, 55] in elite players, while two studies analysed it in school students [36, 37], one study analysed psychological data [43] of the school students, one study analysed health markers [31] in recreational athletes, one study analysed participation in Physical Education class [37], two studies analysed the power of limbs in elite players [58, 59], one study analysed flexibility in elite players [59], one study analysed the indexes volume of play, efficiency and performance score [61], and one study analysed habitual characters of the school students [62]. Therefore, similarly to cross-sectional studies, although we found three to four studies for certain outcomes when analysed for a specific population (e.g., school or university students, or elite players), a maximum of three studies per outcome and population were found.

### Quasi-experimental studies

One cross-sectional study analysed anthropometric characteristics and physical fitness in elite players [24], while two studies analysed skill [24, 53] in elite players [24] and school students [53]. One study analysed knowledge and learning of mini-volleyball in school students [35]. Two studies analysed decision-making [51, 53] in school students [53] and university players [51], and one study analysed tactical-technical behaviour in university players [51]. One study analysed cognitive understanding of content [54] in school students. Two studies analysed skill level, execution, and game performance in school students [57, 60]. One of them analysed game involvement and knowledge besides perceptions of performance [60]. Again, although we found seven studies, when analysed for a specific population or outcome (e.g., university players and decision-making, or university players and tactical-technical behaviour), a maximum of two studies per outcome and population were found.

**FIG. 2:**
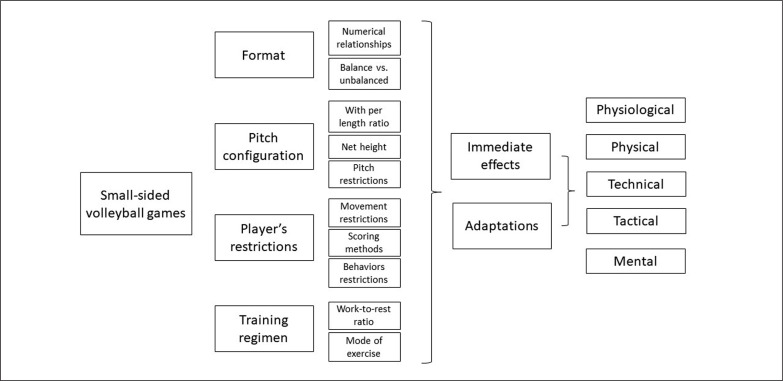
Conceptual map.

In this way, although there was high diversity in the outcomes, there is still a low amount of scientific evidence to be able to make stronger inferences about the effects, efficacy, and effectiveness of SSG in volleyball (Figure 2). This suggests that this is still an area to be profoundly explored in future studies.

### Synthesis methods

No meta-analysis was planned for this study. A narrative synthesis of the results was provided.

## DISCUSSION

The purpose of this systematic review was to summarize and analyse the present state of the art of scientific evidence about SSG in volleyball. According to the obtained results, studies using SSG in volleyball evaluated, in general, the tactical-technical performance (including skill accuracy), decision-making, physiological, physical fitness, and psychological aspects, and health markers in different populations. In addition, studies have used SSG as a way to improve content knowledge and participation in Physical Education classes, and test different instructional approaches in school students and university players, respectively.

### Tactical-technical performance

Two studies [27, 28] applied 2 vs. 2 games in different court sizes to evaluate the participants. When comparing court sizes 3 × 3 m, 4 × 4 m, 4.6 × 4.6 m, and 5.2 × 5.2 m with young novice players, the results showed better adjustment indexes in the 3 × 3 m condition and a better efficiency index in other sizes [28]. Also, higher scores associated with the athlete’s decision-making were found in the 4.6 × 4.6 m and 5.2 × 5.2 m conditions, which demonstrates the importance of increasing the size of the court in the volleyball SSG (area/player ratio). These results demonstrate that smaller dimensions required fewer displacements and facilitated technical performance, considering that volleyball is a non-invasion sport and actions are dependent on previous actions [63, 64]. However, for invasion sports, the literature shows that increased density (less space per player) results in a worse technical index [65].

The study carried out with young elite athletes [28] analysed the games on a 9 m^2^ court, with two different configurations: 3 × 3 m and 2 × 4.5 m. The results show better indexes of adjustment and decision-making in the 2 × 4.5 m configuration for reception and attack. Within the 3 × 3 m condition, on the other hand, the efficacy index was better for reception. Corroborating these results, Gabbett et al. [24] demonstrated improvements in technical skill (spiking and passing) and accuracy in skill tasks (spiking, setting, and passing) using the skill-based conditioning training (3 vs. 3 and 5 vs. 5 players).

However, Gil Arias et al. [53] analysed different dimension courts (6 × 6 m and 4.5 × 4.5 m) and participant numbers (3 vs. 3, 4 vs. 4), and also manipulated the net height to verify the effect on decision-making and attack effectiveness in school students, finding results different from the previous ones, suggesting that tasks for the students who are in the first stages of learning (low level of expertise in decision-making and the effectiveness of technical-tactical actions) should be carried out on wide playing spaces, with smaller participant numbers and with a medium-low net height to detect free spaces in the opposite field and to have a greater distance and longer time between attackers and defenders to favour decisions and action [66].

On the other hand, Gabbett [52] demonstrated that instructional training resulted in considerable improvements in technique and accuracy in all skill tasks (spiking, serving, setting, and passing). Also, Krističević et al. [55] did not find a significant interaction effect on accuracy (overhead pass, forearm pass, setting, serving, and serving under fatigue) of elite young volleyball players between the game-based conditioning group (3 vs. 3 and 4 vs. 4 players) and control group (instructional training) in court size 9 × 4.5 m. Nevertheless, both groups made significant increases in serving accuracy, and the instructional training (control group) induced significant improvements also in forearm passing and setting accuracy, whereas the game-based conditioning group also improved in overhead passing. However, with young female volleyball players, it was found that small-sided games’ training was better in all parameters of accuracy (overhead pass, forearm pass, setting, serving, and serving under fatigue) compared to instructional training [55].

On the other hand, Verscheure and Amade-Escot [56] investigated how the attack is learned in volleyball according to gender in 16 Physical Education students through two tasks involving SSG volleyball (2 vs. 2). The authors concluded that each student constructs the specific knowledge about an efficient attack in volleyball as the result of a complex process related to students’ engagement with the tasks, improving the way of playing (or not) with the learning tasks; cooperation (or not) with their team-mates; and how the teacher’s expectations are expressed through his verbal interactions and the nature of knowledge or values (technical and tactical skills). In another study [35], involving school students, the mini-volleyball programme had a significant impact on the knowledge and learning game situations, along with basic technical skills of 9–11 year-old schoolchildren. Batez et al. [36] compared the Teaching Games for Understanding (TGfU) Mini-volleyball Unit program (experimental group – EG) with traditional Physical Education classes (control group – CG) in adolescent students and after the 6-week intervention the EG improved overhead and forearm passing skills and experienced greater enjoyment compared to the CG. Thus, the SSG (including mini-volleyball) should be included as a fundamental part of Physical Education classes as a process of adaptation to volleyball.

Trying to verify the effects of student skill level (highest and lowest skilled) on knowledge, decision-making, skill execution and game performance, Mahedero et al. [57] used mini-volleyball with 1 vs. 1, 2 vs. 2, 3 vs. 3, and 4 vs. 4 games. The authors found improvements of the higher skilled students in decision-making, but not in skill execution, leading to lower levels of overall game play improvement, while middle skilled students increased in both dimensions, resulting in substantive improvements in game play, and lower skilled students improved in understanding, but were perhaps unable to execute the appropriate skills, resulting in lower gains in game play.

Mahedero et al. [60] explored potential differences in game performance variables and knowledge in high school students with higher or lower skill levels groups. For this, the students had a 12-lesson mini-volleyball sport education unit in which they were submitted to 1 vs. 1, 2 vs. 2, 3 vs. 3, and 4 vs. 4 games. Among the main results, the students became more competent in game play, and more knowledgeable on their technique, the sport’s rules, tactical awareness, and general game understanding. However, grouping students by skill level had no impact on gains in game performance variables and knowledge.

### Physical fitness, physiological aspects and health markers

Gabbett [52] demonstrated, in his study with elite junior’s volleyball players, that skill-based conditioning training (carried out with games of 5 vs. 5, 5 vs. 4, and 5 vs. 3 players) successfully simulated the high-intensity physiological demand of national-level competition, in addition to generating improvements in physical fitness (speed, vertical jump, spike jump, agility, upper-body muscular power, and maximal aerobic power) after training. Similar results were presented by Gabbett et al. [24], who showed improvement in physical fitness (speed and agility) of athletes using skill-based conditioning training (3 vs. 3 and 5 vs. 5 players). This way, Gabbett [52] suggests that a combination of instructional training and skill-based conditioning games is likely to confer the greatest improvements in fitness and skill in junior elite volleyball players. Furthermore, Gjinovci et al. [58] and Idrizovic et al. [59] using skill-based conditioning training (3 vs. 3 and 4 vs. 4 players) found improvement in jumping, sprint, and upper limb strength in elite female volleyball players (adult and juniors, respectively).

Stojanović et al. [8] compared the effect on the body composition in school students of a 16-week programme of volleyball skill-based exercises and SSG (2 vs. 2, 3 vs. 3, and 4 vs. 4) with a regular Physical Education group, revealing more favourable changes in skinfold thickness, body fat tissue, and muscle tissue in the SSG group than the control group, similar for both genders. Another study, carried out by Trajković et al. [31] with middle-aged adults, demonstrated a decrease in some risk factors (specifically LDL, resting HR, and systolic BP) and better results in cardiovascular fitness after 10 weeks of small-sided recreational volleyball (3 vs. 3 and 4 vs. 4 players) in court size 16 × 8 m.

Studies in soccer demonstrated that the SSG intensity is higher on larger pitches and with a smaller number of players [30]. However, although the studies in soccer [29, 32] show different intensities with different SSG manipulations, and our understanding that it can influence physical and physiological variables, we do not have specific data in volleyball for a more in-depth discussion of this correlation.

### Psychological aspects

A study by Trajković et al. [43] demonstrated that 8 months of small-sided volleyball training (3 vs. 3 and 4 vs. 4 players in court sizes 9 × 4.5 m and 12 × 6 m) twice a week after school in addition to the regular Physical Education classes was able to significantly decrease adolescent aggression and presented better results in physical fitness compared to Physical Education classes only. Studies using a mini-volleyball programme with children [35] and adolescent school students [36], after the 9-month and 6-weeks interventions, respectively, showed an improvement in the motivation to sport practice [35, 36] and enjoyment in Physical Education classes [36].

### SSG with different instructional approaches

Regarding this topic, Kim [54] evidenced, among other aspects, that when exchanging the formal game (6 vs. 6) for the use of SSG (3 vs. 3 and 4 vs. 4) the school students had impactful changes in game performance (e.g., more control over technique, higher success rate, and less peer pressure), involvement, as well as a cognitive understanding of content.

To test different instructional approaches, Broek et al. [51] analysed the decision-making process of university students divided into three instructional groups (teacher-centred, student-centred with and without tactical questioning) using SSG (4 vs. 4). After the intervention period the tactical awareness of all students improved and this effect persisted over time. After six weeks, male students showed significantly higher tactical awareness scores than females, and the volleyball-specific tactical knowledge of the student-centred instructional group with tactical questioning improved significantly more compared with the two other instructional groups.

In this context, Sgrò et al. [61] examined the effects of a tactical games model instructional plan on game-play volleyball performances of elementary school students, taking into account their skill level. In total, 39 elementary school students (average age: 8.9 years) participated in a 13-week unit, in which each lesson exaggerated the use of SSG. At the end of the instructional period, all participants had an overall moderate to large improvement, and this global improvement seems to have remained at least until the end of the summer vacation. Lower-skilled students attained a larger and more established improvement than high-skilled students did. However, some detrimental effects on in-game students’ performance existed at the end of the instructional period. Therefore, teachers have to take into account students’ skill levels when designing their lessons because, if SSG are adequately considered and managed, students’ learning processes can be enhanced.

Sujarwo et al. [62] noted that all of the Physical Education teachers in elementary school focus on volleyball skill and knowledge in their teaching. In this way, when using the mini-volleyball learning model, this approach allowed students to display characteristics of discipline, cooperation, and hard work. Additionally, a recent study by Ningrum et al. [37] demonstrated that high school students who experienced the SSG learning model on the mobile phone application had higher activity levels and interest in Physical Education, socialization, and volleyball forearm pass skill. Despite the need for more studies, this can be a tool to increase student interest in volleyball.

### Study limitations, future research, and practical applications

Despite the innovative information on the effects of using SSG in volleyball, some key points are worth mentioning. Although most of the studies presented have high methodological quality, there were a few modifications used to evaluate the effects of small games in volleyball, summarized by changes in the number of athletes (1 vs. 1 to 5 vs. 5) in most studies, net height (1.60 m and 2.18 m), and the dimensions of the court (2 × 4.5 m to 12 × 6 m), therefore investigating SSG in volleyball in the court. In addition, we did not include specific teaching models in our search, limiting the results. This is a limitation of the study and a suggestion for future research analysis.

We highlight that there is a lack of information about SSG in volleyball, different from what we found, for example, in soccer or basketball (see Clemente et al. [10]). Various study designs (different court dimension, number of players, rules and duration modification, etc.) provide a broader knowledge about the possible manipulations of the performance variables [25], but the inconsistencies between studies, and the different sample sizes, make it difficult to extrapolate the results in volleyball. Furthermore, few studies used SSG in volleyball, particularly with high-performance adult athletes. At this point, future studies in volleyball should carry out other modifications of small games with different populations to evaluate their effects, including the association between SSG and intensity. Another point is the lack of information on rule manipulation, functional specialization, and the outcomes of long-term use of volleyball SSG, which should also be explored.

With this study, we hope that teachers, coaches, and people involved with volleyball use the SSG as a tool for the development and assessment of their students/athletes. In addition, we hope volleyball practitioners will use this approach for tactical-technical and physical development. Finally, we hope to encourage Sports Science researchers to conduct studies using SSG in volleyball, as this is necessary and promising research.

## CONCLUSIONS

Based on the results of this systematic review, we conclude that there is evidence supporting the pedagogical and training use of SSG in volleyball and its positive effects in populations of different levels of training as outcomes of anthropometric characteristics (school students), cognitive understanding of content and sport knowledge (school students), decision-making (university students, school students, and young athletes), health markers (recreational adult players and school students), instructional approaches (university students), physical fitness, psychological, attack efficacy (school students), skill accuracy, and tactical-technical behaviour (young and adult athletes). However, the particular constraints put in place by each specific SSG should be further explored to provide coaches and Physical Education teachers with more information on how to design SSG that are appropriate for different goals. Thus due to great methodological and sample heterogeneity used in studies, the present results should be interpreted with caution. Furthermore, when analysing the studies, we concluded that despite the limited evidence for each outcome, and the absence of cause-and-effect studies (preferably, randomized trials), the results can help coaches, as well as Physical Education and sports professionals, to plan tasks during their intervention and training to optimize the performance of players.
